# Clinical characteristics of pneumorrhachis in dogs and cats: a retrospective study of 125 cases

**DOI:** 10.1093/jvimsj/aalag250

**Published:** 2026-10-09

**Authors:** Sam Ooms, Kenny Bossens, Ine Cornelis, Iris van Soens, Kaatje Kromhout, Sofie F M Bhatti

**Affiliations:** Small Animal Department, Faculty of Veterinary Medicine, Ghent University, 9820 Merelbeke- Melle, Belgium; Nesto Veterinary Clinic Orion, 2200 Herentals, Belgium; Nesto Veterinary Clinic Orion, 2200 Herentals, Belgium; Small Animal Department, Faculty of Veterinary Medicine, Ghent University, 9820 Merelbeke- Melle, Belgium; IVC Evidensia Small Animal Referral Hospital Hart van Brabant, 5144 AM Waalwijk, The Netherlands; Department of Morphology, Imaging, Orthopedics, Rehabilitation and Nutrition, Faculty of Veterinary Medicine, Ghent University, 9820 Merelbeke- Melle, Belgium; Small Animal Department, Faculty of Veterinary Medicine, Ghent University, 9820 Merelbeke- Melle, Belgium

**Keywords:** intraspinal gas, vacuum phenomenon, intervertebral disc disease, neurological diseases

## Abstract

**Background:**

Pneumorrhachis (PR), defined as gas within the vertebral canal, is an uncommon finding, and its clinical relevance and relationship to the vacuum phenomenon (VP) have not been systematically evaluated.

**Hypothesis/Objectives:**

To characterize the anatomical distribution of PR in dogs and cats undergoing computed tomography (CT) and to assess its relationship with signalment, VP, and concurrent neurological and non-neurological conditions.

**Animals:**

One hundred nineteen dogs and 6 cats with PR identified on CT between 2015 and 2023.

**Methods:**

Retrospective, descriptive study of medical records and CT reports. Cases were included when PR was documented in the radiology report, clinical and imaging information was available. Data included signalment, PR distribution, VP presence, final diagnosis categorized as neurological, non-neurological, postoperative, or no identified cause.

**Results:**

Pneumorrhachis was identified with neurological conditions in 60/119 dogs (50%), including intervertebral disc disease (IVDD) in 50/60 (83%). Among IVDD-related PR, Hansen type I occurred in 32/50 (64%) cases. A VP was present in 23/50 dogs (46%), co-localized with the affected intervertebral disc in 78%. Non-neurological conditions accounted for 45/119 (38%) dogs, mainly neoplastic (44%) and respiratory (36%), with VP infrequently detected. Pneumorrhachis occurred postoperatively in 12/119 dogs. Two dogs (2%) had PR and epaxial muscle pain without an identifiable cause. In cats, PR was associated with neurological (2/6), non-neurological conditions (2/6), and postoperatively (2/6). No cases involved IVDD or VP.

**Conclusions and clinical importance:**

Pneumorrhachis in dogs and cats is considered an uncommon CT finding occurring with neurological and non-neurological conditions. Although often accompanying IVDD in dogs, PR was frequently incidental.

**Abstract (Dutch):**

**Achtergrond**

Pneumorrhachis (PR), gedefinieerd als de aanwezigheid van gas in het wervelkanaal, wordt beschouwd als een zeldzame bevinding, hoewel PR is beschreven in verband met intervertebrale discusdegeneratie (IVDD), zijn de klinische relevantie en de relatie met het vacuümfenomeen (VP) niet systematisch geëvalueerd.

**Doelstellingen**

Het karakteriseren van de anatomische verdeling van PR bij honden en katten die computer tomografie (CT) ondergingen en het beoordelen van de relatie met signalement, VP en gelijktijdig voorkomende neurologische en niet-neurologische aandoeningen.

**Dieren**

119 honden en 6 katten met PR, vastgesteld op CT tussen 2015 en 2023.

**Methoden**

Retrospectieve, beschrijvende studie van medische dossiers en CT-verslagen. Cases werden geïncludeerd wanneer PR geschreven werd in het radiologie verslag en wanneer alle klinische en beeldvorming informatie beschikbaar was.

**Resultaten**

Pneumorrhachis werd vastgesteld bij 60/119 honden (50%) met neurologische aandoeningen, waaronder IVDD bij 50/60 (83%). Van de gevallen met IVDD-gerelateerde PR hadden 32/50 (64%) een Hansen type I. Een VP was aanwezig bij 23/50 honden (46%) en bevond zich bij 78% ter hoogte van de aangetaste tussenwervelschijf. Niet-neurologische aandoeningen waren aanwezig bij 45/119 (38%) honden, voornamelijk neoplastische (44%) en respiratoire aandoeningen (36%), waarbij VP slechts zelden werd vastgesteld. Pneumorrhachis trad postoperatief op bij 12/119 honden. Twee honden (2%) hadden PR en epaxiale spierpijn zonder identificeerbare oorzaak. Bij katten was PR geassocieerd met neurologische aandoeningen (2/6), niet-neurologische aandoeningen (2/6) en een postoperatieve toestand (2/6). Geen van de katten had IVDD of VP.

**Conclusies en klinische relevantie**

PR bij honden en katten wordt beschouwd als een zeldzame CT-bevinding die voorkomt bij zowel neurologische als niet-neurologische aandoeningen. Hoewel PR bij honden vaak gepaard gaat met IVDD, is het vaak een incidentele bevinding.

## Introduction

Pneumorrhachis (PR), the presence of gas within the vertebral canal, is considered an uncommon finding in veterinary and human medicine.^1–3^ Pneumorrhachis might occur in the extradural or intradural space and is usually composed of nitrogen-rich gas.^4^ Although occurring in association with a variety of clinical conditions, the mechanisms underlying gas accumulation in the vertebral canal remain incompletely understood.^1^^,^^2^^,^^5^

The veterinary literature on PR remains limited and consists predominantly of single case reports or small case series. Pneumorrhachis in dogs and cats is reported most commonly in dogs with intervertebral disc disease (IVDD) and an associated vacuum phenomenon (VP)^5^^,^^6^; however, iatrogenic,^2^^,^^7–10^ traumatic,^11^^,^^12^ and infectious etiologies^13^ is described.^3^ Iatrogenic PR can occur secondary to intravenous (IV) contrast injections, locoregional anesthetic techniques, or spinal surgical procedures.^3^^,^^7^ Pneumorrhachis associated with the VP is characterized by the accumulation of gas within the intervertebral disc space or vertebral body. In such cases, gas originating from a degenerated disc can migrate into the vertebral canal through annular fissures or structural defects.^5^^,^^6^^,^^14^ Alternatively, gas might enter the subarachnoid space along pressure gradients generated by cerebrospinal fluid leakage.^2^

Similar to observations in human medicine, PR in dogs and cats is often considered an incidental imaging finding without clinical relevance.^1^^,^^2^ Nevertheless, isolated case reports have documented clinically relevant PR associated with spinal pain or neurological deficits.^5^^,^^15^ Reported associations include compressive PR following surgery for IVDD^2^, craniotomy^8^^,^^10^ and rhinotomy,^9^ as well as cases associated with a VP^15^ or head trauma.^11^^,^^12^ In these cases, PR has been regarded as clinically relevant due to mass effect or potential chemical irritation of neural structures. Reports in cats remain rare and typically describe isolated spontaneous or iatrogenic causes.^7^^,^^13^

In human medicine, computed tomography (CT) is considered the most sensitive technique for the detection of PR, and it is valuable in veterinary patients.^2^^,^^10^^,^^12^ Magnetic resonance imaging (MRI) allows evaluation of potential coexisting etiologies but is less reliable for the detection of PR because of the difficulty in distinguishing gas from other signal voids.^1^

The objective of this retrospective, descriptive study was to characterize PR in dogs and cats undergoing CT imaging, and to identify its anatomical distribution, to evaluate the presence and location of a VP, and to determine concurrent neurological and non-neurological conditions.

## Materials and methods

The medical records and CT reports of all dogs and cats that underwent a CT-examination of any portion of the vertebral column between 2015 and 2023 at the Small Animal Department (Faculty of Veterinary Medicine, Ghent University, Belgium) and at the Veterinary Clinic Orion (Herentals, Belgium) were retrospectively reviewed. Cases were eligible for inclusion when “pneumorrhachis” was documented in the original radiology report and when complete clinical and imaging information was available. Pneumorrhachis was recorded as described in the initial CT report; no systematic re-evaluation of CT images was performed for the purpose of this study. Imaging studies were reviewed by a radiology resident supervised by a board-certified radiologist.

For each animal, the following variables were extracted from the medical records: signalment (species, breed, sex, age), anatomical distribution of PR, presence and location of VP, and final diagnosis, categorized as neurological condition (IVDD or other spinal disorders), non-neurological condition, postoperative PR, or PR without an identifiable underlying cause.

Computed tomography examinations at Ghent University were performed using a 320-slice single-source CT scanner (Aquilion ONE VISION, Canon Medical Systems), whereas examinations at the Veterinary Clinic Orion were performed using a 16-slice CT scanner (Toshiba Aquilion RXL). Acquisition parameters varied depending on patient size and clinical indication, but all studies included transverse images with multiplanar reconstructions.

This study used only pre-existing clinical data and imaging records; therefore, ethical approval was not required. All data were anonymized before analysis.

## Results

A total of 119 dogs and 6 cats met the inclusion criteria. Of these, 109 dogs and 5 cats were examined at the Small Animal Department of Ghent University, and 10 dogs and 1 cat at the Veterinary Clinic Orion. The mean age at presentation was 7.5 years (range, 0.7-17.8 years) in dogs, whereas cats presented at a mean age of 6.5 years (range, 0.8-15 years). In dogs, 39% (46/119) were intact males, 23% (27/119) were neutered males, 14% (17/119) were intact females, and 24% (29/119) were neutered females. Among the cats, 66% (4/6) were neutered males, 17% (1/6) were intact females, and 17% (1/6) were neutered females. In 60 of 119 dogs (50%), PR was associated with neurological conditions (Figure 1). Among these, 50 dogs (83%) had IVDD, and 10 dogs (17%) were diagnosed with other spinal disorders. In dogs with IVDD-associated PR, Hansen type I was diagnosed in 32 of 50 dogs (64%), Hansen type II in 13 of 50 dogs (26%), hydrated nucleus pulposus extrusion (HNPE) in 2 of 50 dogs (4%), acute non-compressive nucleus pulposus extrusion (ANNPE) in 2 of 50 dogs (4%), and degenerative lumbosacral stenosis (DLSS) in 1 of 50 dogs (2%). The spinal cord segments affected in IVDD- associated PR cases included T3–L3 in 35 dogs (70%), L4–S3 in 11 dogs (22%), C6–T2 in 3 dogs (6%), and C1–C5 in one dog (2%). In 33 of 50 dogs (66%) with IVDD-associated PR, the localization of the PR corresponded to the same affected intervertebral disc space, and these cases were diagnosed with Hansen type I (21/33, 64%), Hansen type II (10/33, 30%), and HNPE (2/33, 6%). In 23/50 dogs (46%) a VP was present, mostly associated with Hansen type I (11/23, 48%), Hansen type II (10/23, 44%), and less frequently seen with HNPE (1/23, 4%) and DLSS (1/23, 4%). In 18 of these 23 dogs (78%), the VP was located at the same site as the affected intervertebral disc space. Among the 10 dogs with non-IVDD spinal disorders, PR occurred within L4–S3 in 4 dogs (40%), T3–L3 in 3 dogs (30%), C6–T2 in 2 dogs (20%), and C1–C5 in 1 dog (10%). A VP was noted in these 3 (30%), and in one of these 3 (33%) the VP corresponded to the site of the spinal lesion.

**Figure 1:**
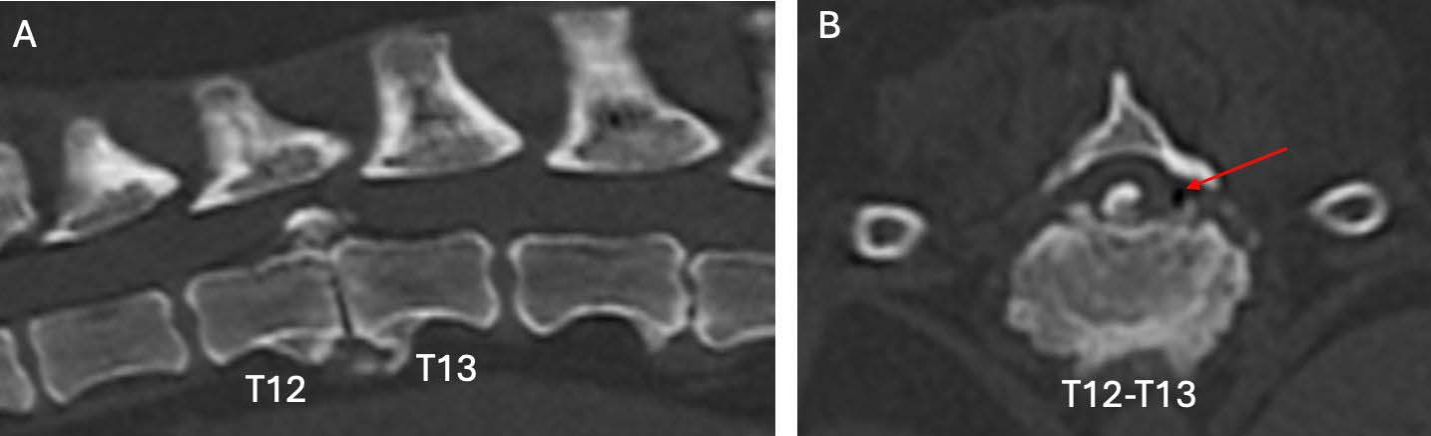
Computed tomographic images of a dog with IVDD-related PR at the intervertebral disc space T12-T13. (A) Sagittal computed tomography reconstruction, bone window. (B) Transverse computed tomography, bone window. Red arrow, pneumorrhachis.

In 45 of 119 dogs (38%), non-neurological conditions were identified and consisted of neoplastic diseases in 20 dogs (44%), respiratory diseases in 16 dogs (36%), and various other disorders in 9 dogs (20%) (Figure 2). In dogs with neoplastic disease, PR was most commonly located between the T1 and T6 vertebral body levels (7/20, 35%) and at the C7–T1 intervertebral disc space (3/20, 15%). A VP was observed in 4 of 20 dogs (20%) with neoplasia and was located at L7–S1 intervertebral disc space in 2 dogs (50%), L5–L6 in one dog (25%), and L6–L7 in one dog (25%). In 3 of these 4 dogs (75%), VP and PR occurred at the same site. In the respiratory disease subgroup, PR was located in the cervicothoracic region in 15 of 16 dogs (94%), and no VP was identified. In dogs with other non-neurological conditions, PR was most frequently detected at T5–T6 (4/9, 44%), and similarly, no VP was recorded.

**Figure 2:**
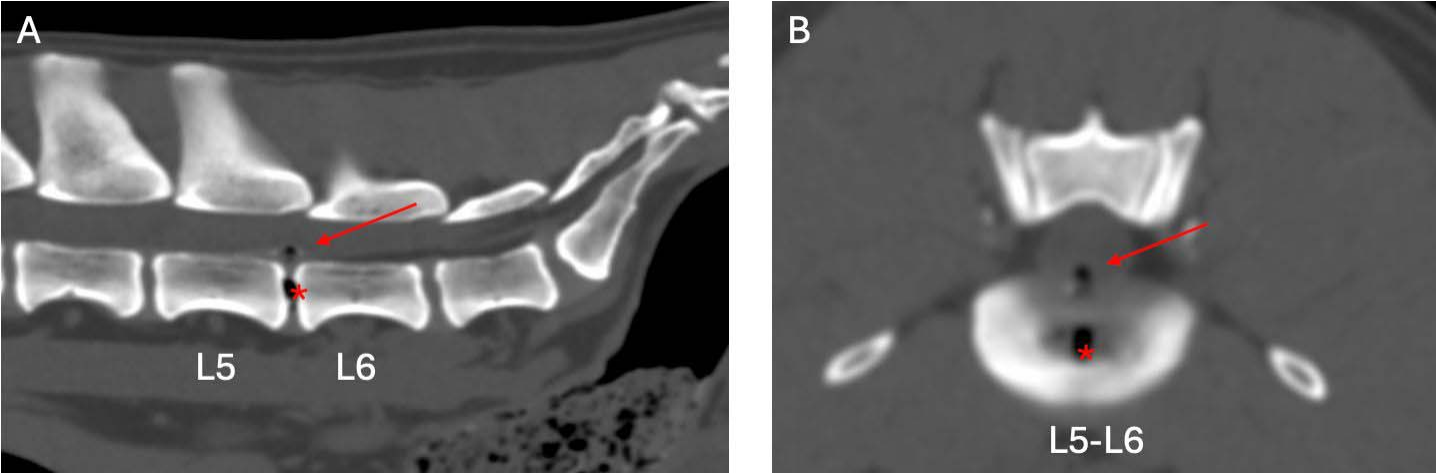
Computed tomographic images of a dog with non- neurological condition (prostatic carcinoma) with PR and VP. (A) Sagittal computed tomography, bone window. Red asterisk, vacuum phenomenon; red arrow, pneumorrhachis. (B) Transverse computed tomography, bone window. Red asterisk, vacuum phenomenon; red arrow, pneumorrhachis.

In 12 of 119 dogs (10%), PR occurred after a spinal surgical procedure (Figure 3). In all cases, PR was located at the same intervertebral disc level as the surgical site. A VP was identified in only one of these dogs (8%). Two of 119 dogs (2%) presented with spinal pain without identification of an underlying cause (Figure 4). In these dogs, PR was located at the T6 vertebral body in one dog, which exhibited pain on palpation of the epaxial muscles, and within the C6–T2 spinal cord region in the other dog, which exhibited pain on palpation of the cervical region. No VP was observed. In 2 of 6 cats (33%), PR was associated with neurological conditions. One cat was diagnosed with constrictive myelopathy secondary to pia-arachnoid fibrosis following a hemilaminectomy and had PR in the thoracolumbar region, whereas the other had multiple skull fractures with PR at C1. No cases of IVDD or VP were identified in neurologically affected cats. Non-neurological conditions were diagnosed in 2 cats (33%): one cat with nasopharyngeal stenosis secondary to chronic inflammation had PR in the thoracic region, and one cat with metastatic pancreatic adenocarcinoma had PR at L7–S1. No VP was present in this group. The remaining 2 cats (33%) developed postoperative PR, as no PR was identified on the preoperative CT examination. One cat underwent hemilaminectomy for articular process hypertrophy and the other underwent vertebral stabilization of a T13–L1 fracture–luxation. In both cats, PR was located at the surgical site, and no VP was identified.

**Figure 3:**
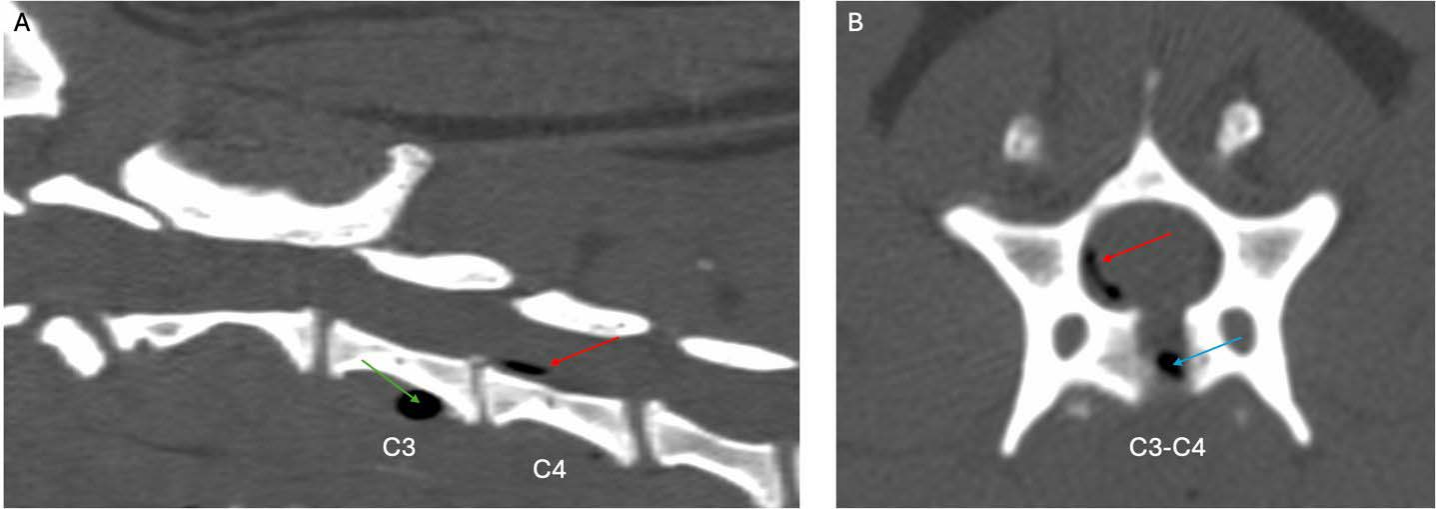
Postoperative computed tomographic images of a dog following a ventral slot procedure C3-C4. (A) Parasagittal computed tomography reconstruction, bone window. Red arrow, pneumorrhachis. Green arrow, extravertebral gas in the soft tissue surroundings. (B) Transverse computed tomography, bone window. Red arrow, pneumorrhachis. Blue arrow, air within the surgical defect outside the vertebral canal.

**Figure 4:**
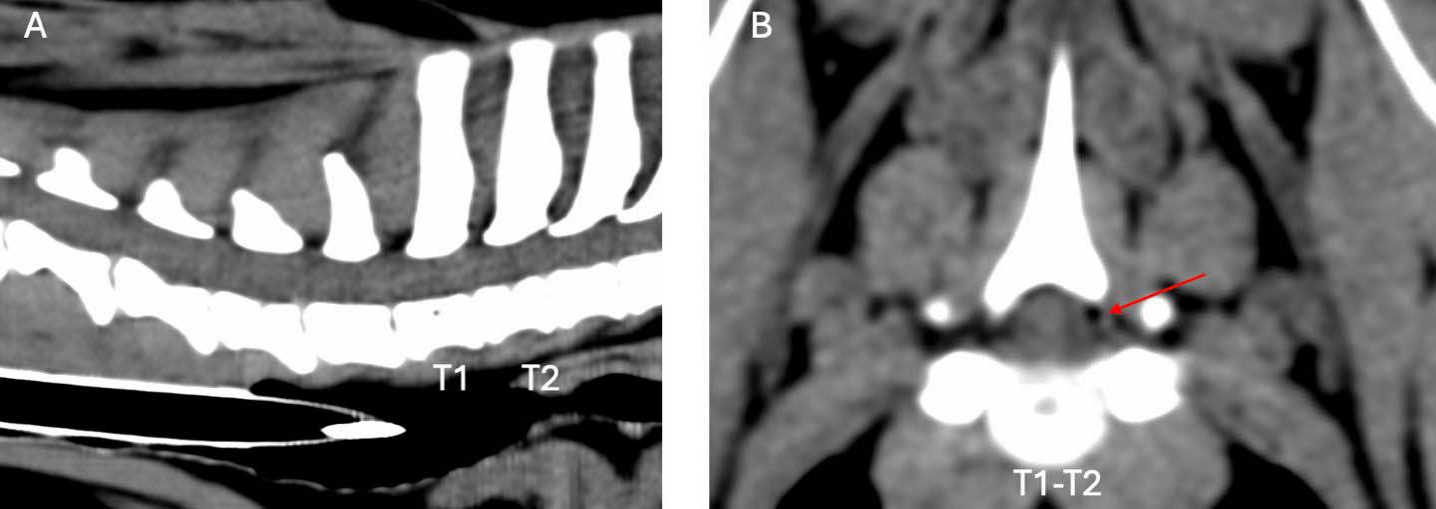
Computed tomographic images of a dog with pain on palpation of the epaxial muscles only with PR. (A) Sagittal computed tomography reconstruction, soft tissue window. (B) Transverse computed tomography, soft tissue window. Red arrow, pneumorrhachis.

## Discussion

This retrospective study describes the clinical characteristics of PR in dogs and includes a small series of cats. Previous veterinary reports largely consist of isolated case reports or small series, where PR was most commonly associated with IVDD,^5^^,^^6^ iatrogenic,^2^^,^^7–10^ traumatic,^11^^,^^12^ spontaneous and infectious diseases.^13^ In contrast, the present study demonstrates that PR occurs across a wide spectrum of neurological and non-neurological conditions. The large and heterogeneous cohort included in our study allowed a more comprehensive evaluation of PR distribution patterns and clinical associations in dogs and cats.

In our study, when PR was visible on a CT study of a dog, it was associated with IVDD in 50% of the cases, suggesting that IVDD might be the most common neurological condition associated with PR. A VP was detected in fewer than half of IVDD-associated PR cases, but when present, VP was usually co-localized with the affected disc. These findings support the proposed mechanisms in which gas migrates into the vertebral canal through annular ruptures or microstructural defects that connect intradiscal gas with the epidural space.^7^^,^^14^^,^^16^ However, the absence of VP in many affected dogs suggests that additional mechanisms—such as cerebrospinal fluid leakage creating negative pressure gradients, subtle annular tears, or biomechanical factors related to body posture and movement—might also contribute to gas accumulation within the vertebral canal.^2^

Importantly, 38% of the reported dogs with PR had non-neurological conditions and PR was often localized anatomically remote from the primary pathology. This observation suggests that PR in these cases was likely incidental. In dogs undergoing CT primarily for oncologic screening, PR was visible on both pre- and postcontrast images in 56% of cases, whereas 44% had PR identified only before or after contrast administration, which supports the theory of an iatrogenic source.^3^ In dogs with respiratory disease, PR was typically located in the cervicothoracic region, a distribution pattern consistent with retrograde migration of mediastinal air through neural foramina or vascular pathways, as described in human medicine.^1^^,^^14^ Neoplastic disease represented a substantial proportion of non-neurological cases, with PR most frequently localized to the cranial thoracic spine. This distribution is consistent with previous findings, with C7–T1 and the mid-thoracic vertebral column as common sites of PR.^3^ An alternative explanation could relate to regional venous anatomy. The third to fifth dorsal intercostal veins form a common venous trunk that drains via the sixth dorsal intercostal vein into the azygos vein, potentially providing an anatomical route for air migration.^3^ Pneumorrhachis developing after spinal surgery accounted for a smaller proportion of cases and was consistently localized at the surgical site. The low prevalence of a VP in this subgroup supports the interpretation that postoperative PR is primarily iatrogenic, resulting from intraoperative introduction of air during exposure of the vertebral canal. In agreement with previous reports in both human and veterinary literature, these cases were not consistently associated with clinically relevant deterioration, suggesting that postoperative PR is generally self-limiting without clinical relevance.^2^^,^^5^

In most dogs of our study in which PR was identified, clinical signs were attributable to the underlying disease process, suggesting that PR was unlikely to contribute additional clinical signs. However, two dogs exhibited spinal pain without identification of an underlying cause. One dog had pain on palpation of the epaxial muscles, whereas the other dog exhibited pain on palpation of the cervical region as the sole clinical sign. In these cases, no structural spinal or extraspinal abnormalities were detected on CT apart from PR, and VP was not identified in either dog. Pneumorrhachis was located at T6 in one dog and within the C6–T2 region in the other. These observations suggest that PR can occur in the absence of demonstrable compressive or infiltrative disease. Although causality cannot be inferred, several pathophysiological mechanisms could plausibly explain pain in the context of PR without a detectable lesion on CT. Epidural gas might exert transient focal mass effect or chemical/mechanical irritation of nociceptive structures (eg, meninges, periarticular soft tissues, dorsal longitudinal ligament), leading to hyperesthesia.^1^^,^^5^ The clinical significance of PR in these cases cannot be determined with certainty. To better establish a potential causal relationship, longitudinal follow-up would be required. Specifically, follow-up CT imaging after resolution of clinical signs could help determine whether disappearance of PR correlates with clinical improvement, thereby supporting its clinical relevance in such cases. Cats represented a small proportion of the study cohort, and no IVDD-associated PR was identified, consistent with the lower prevalence of clinically relevant IVDD in this species.^17^^,^^18^ Although limited numbers preclude species-specific conclusions, PR in cats appeared to arise in comparably diverse clinical contexts as in dogs, including neurological, non-neurological, and postoperative conditions.

Clinically, the detection of PR—particularly when accompanied by a VP—should prompt careful evaluation of intervertebral discs for degenerative lesions. However, PR should not be interpreted as inherently indicative of spinal cord dysfunction, as it occurred across a wide range of neurological and non-neurological conditions and was most frequently incidental. The presence of PR in cases with respiratory or neoplastic disease highlights the importance of considering extravertebral sources of gas migration.

Several limitations should be acknowledged. The retrospective design introduces a potential selection, reporting, and documentation bias, particularly because only cases with PR explicitly recorded in radiology reports were included. Imaging protocols were not standardized across facilities, and detailed localization data were incomplete for some non-neurological cases. Outcome data were not available, limiting assessment of the prognostic relevance of PR. Finally, causality could not be inferred from this descriptive study.

## Conclusions

Our results demonstrated that PR was most frequently identified in dogs and was strongly associated with IVDD, particularly Hansen type I. In dogs, PR was also common with non-neurological conditions—most notably respiratory and neoplastic conditions—where it was anatomically unrelated to the primary lesion. In cats, PR occurred less frequently and in heterogeneous contexts, reflecting species-specific differences in spinal disease prevalence.

Overall, these findings demonstrate that PR is not a diagnosis. When detected, PR warrants careful evaluation of intervertebral discs and consideration of both spinal and extra-spinal causes. Because PR often lacks clinical relevance, its interpretation must be guided by the broader clinical picture, including neurological examination, imaging findings, and underlying disease processes.

## Off-label antimicrobial declaration

Authors declare no off-label use of antimicrobials.

## Institutional animal care and use committee or other approval declaration

Authors declare no institutional animal care and use committee or other approval was needed.

## Abbreviations

CT: computed tomography
IVDD: intervertebral disc disease
MRI: magnetic resonance imaging
PR: pneumorrhachis
VP: vacuum phenomenon
